# The relevance of microRNA in post-infarction left ventricular remodelling and heart failure

**DOI:** 10.1007/s10741-019-09770-9

**Published:** 2019-02-02

**Authors:** Mieczysław Dutka, Rafał Bobiński, Jan Korbecki

**Affiliations:** Department of Biochemistry and Molecular Biology, University of Bielsko-Biala, Faculty of Health Sciences, Willowa St. 2, 43-309 Bielsko-Biała, Poland

**Keywords:** MicroRNA, Myocardial infarction, Left ventricular remodelling, Myocardial fibrosis, Heart failure

## Abstract

Myocardial infarction and post-infarction left ventricular remodelling involve a high risk of morbidity and mortality. For this reason, ongoing research is being conducted in order to learn the mechanisms of unfavourable left ventricular remodelling following a myocardial infarction. New biomarkers are also being sought that would allow for early identification of patients with a high risk of post-infarction remodelling and dysfunction of the left ventricle. In recent years, there has been ever more experimental data that confirms the significance of microRNA in cardiovascular diseases. It has been confirmed that microRNAs are stable in systemic circulation, and can be directly measured in patients’ blood. It has been found that significant changes occur in the concentrations of various types of microRNA in myocardial infarction and heart failure patients. Various types of microRNA are also currently being intensively researched in terms of their usefulness as markers of cardiomyocyte necrosis, and predictors of the post-infarction heart failure development. This paper is a summary of the current knowledge on the significance of microRNA in post-infarction left ventricular remodelling and heart failure.

## Introduction

Despite significant progress in the treatment of cardiovascular diseases, they continue to be the principal cause of death in developed countries. This is mainly due to acute coronary syndromes. The current methods used in the treatment of myocardial infarction patients have significantly lowered early mortality from this disease. However, many myocardial infarction patients suffer from unfavourable left ventricular remodelling and the development of heart failure. Although there have been improvements in its treatment, heart failure remains a very serious clinical problem. It causes high mortality, and many patients suffer from severe symptoms despite optimal treatment. For this reason, efforts are being made both to understand the mechanisms that determine unfavourable left ventricular remodelling and the development of heart failure in some myocardial infarction patients, and to learn about the protective mechanisms that safeguard other patients from such remodelling.

Many different studies have confirmed the instance of significant changes in the concentrations of cytokines and soluble tumour necrosis factor (TNF) family receptors in the plasma of patients in the acute phase of myocardial infarction [1–6]. These changes are seen, among others, in TNF-alpha, osteoprotegerin (OPG) and the TNF-related apoptosis-inducing ligand (TRAIL) [2, 5]. A high concentration of OPG in plasma, especially a high OPG/TRAIL ratio, has been confirmed by research to be a powerful predictor of cardiovascular mortality in myocardial infarction patients, both ST segment elevation myocardial infarction (STEMI) and non-ST segment elevation myocardial infarction (NSTEMI) [4, 6]. It has been shown that higher post-myocardial infarction mortality in patients with high concentrations of OPG and high OPG/TRAIL ratios is related mainly to unfavourable left ventricular remodelling and the development of heart failure after a myocardial infarction. In addition to the above-mentioned cytokine and TNF family receptors, there is a growing interest in bone marrow-derived stem and progenitor cells, including mesenchymal stem cells (MSC). These are mobilised from bone marrow, for example during acute myocardial infarction [5, 7, 8]. The degree of this mobilisation and the subsequent amount of mesenchymal stem cells in peripheral blood vary. The mechanisms of this diversity are not fully understood.

The presence of a smaller number of MSC in the peripheral blood in the acute phase of myocardial infarction has been demonstrated in patients who go on to develop unfavourable left ventricular remodelling and heart failure when compared with those patients in whom this does not occur [5]. These observations, among others, have become the basis for research and tests into the mechanisms regulating the proliferation and variation of bone marrow-derived stem and progenitor cells. One of the areas of this research is related to the testing of microRNA (miRNA) expression, which, regulates, among other things, the proliferation and variation of stem cells. Additionally, some of the numerous paracrine factors released by the MSC conduct their biological action by regulating the expression of miRNAs. MiRNAs are additionally related to the regulation of the processes of cardiomyocyte apoptosis and fibrosis within the heart. These are basic processes involved in post-infarction left ventricular remodelling and the development of heart failure.

## Structure and origin of microRNA

This is a group of small, non-coding RNA molecules which regulate the expression of genes at the transcriptional and post-transcriptional stages. MiRNAs are the most numerous group of small regulatory RNAs, known as srRNAs. SrRNAs include miRNAs, which take part in gene silencing at the post-transcriptional or transcriptional stage, small interfering RNAs (siRNAs), which silence genes at the post-transcriptional stage, and trans-acting siRNAs, anti-sense iRNAs and pivi-interactive RNAs [9]. MicroRNAs are known by the abbreviation miRNAs, or more often miRs, to which appropriate identification numbers are added appropriate to the type of microRNA. If the same miRNAs are formed from two precursors, this is indicated by the addition of numbers, e.g. miR–218-1 and miR–218-2. If the microRNAs differ only slightly in their nucleotide sequence, this is indicated by the addition of letters, e.g. miR–208a and miR–208b. The name of microRNA molecules can also contain information about the branch they belong to e.g. iR-499-5p [10]. MiRNAs have been identified in vertebrates, insects, plants and fungi, as well as in bacteria and viruses. SiRNAs have been found in animals, plants, fungi and ciliates [11]. To date, over 2000 miRNAs have been identified in humans [12–14]. These regulate 30% of the human genome. It is thought that one type of miRNA may be responsible for the expression of many genes. Additionally, individual genes may be regulated by several different microRNA molecules [13].

**Fig. 1:**
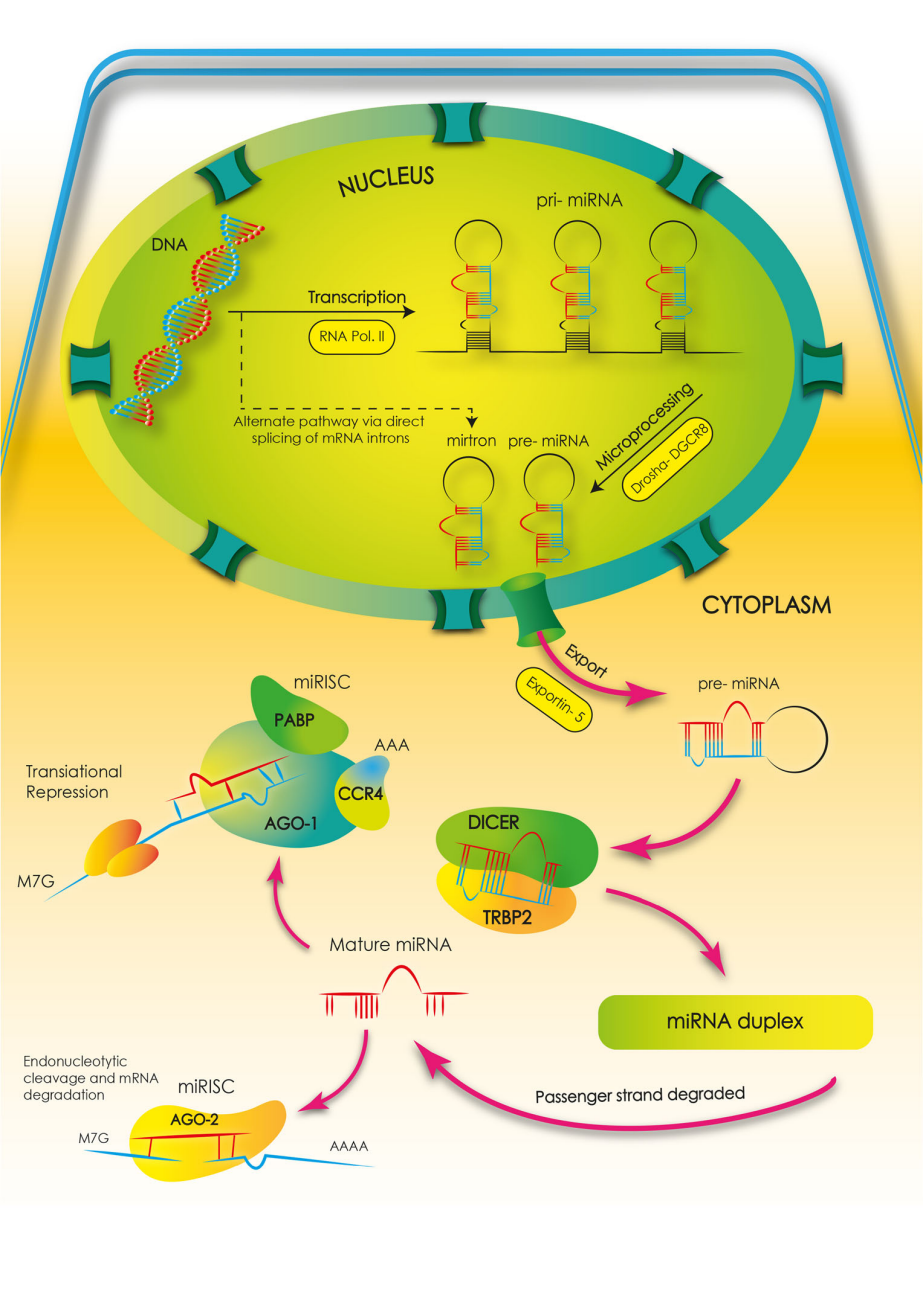
MicroRNA biogenesis. The first stage of biogenesis of microRNA is the transcription of miRNA genes performed in the nucleus with the participation of RNA polymerase II. This process results in the creation of pri-miRNAs. At the next stage, the pri-miRNAs are cleaved into precursor miRNAs (pre-miRNAs). This happens with the participation of the Drosha enzyme in association with the DGCR8 protein (DiGeorge syndrome Critical Region 8). This complex creates pre-miRNA. There is also an alternative pathway by the direct splicing of mRNA introns, bypassing the Drosha processing. The pre-miRNA molecules formed in this way are called mirtrons. Both these types of molecules (pre-miRNAs and mirtrons) are actively transported to the cytoplasm by Exportin-5. In the cytoplasm, the next stage of the maturation of pre-miRNA takes place. At this stage, the Dicer enzyme together with TRBP2 (transactivating response RNA-binding protein) creates the double-stranded miRNA duplexes. Each of these duplexes has a functional miRNA leading strand and a passenger strand. Next, the duplex is unwound, and the passenger strand is degraded. From this moment, the leading strand, as a mature miRNA, can enter the miRISC (miRNA-induced silencing complex) by connecting with Argonaute proteins. Other abbreviations: *PABP* poly A binding protein, *CCR4* C-C chemokine receptor type 4, *AGO*-1 Argonaute protein 1, *AGO*-2 Argonaute protein 2

**Fig. 2:**
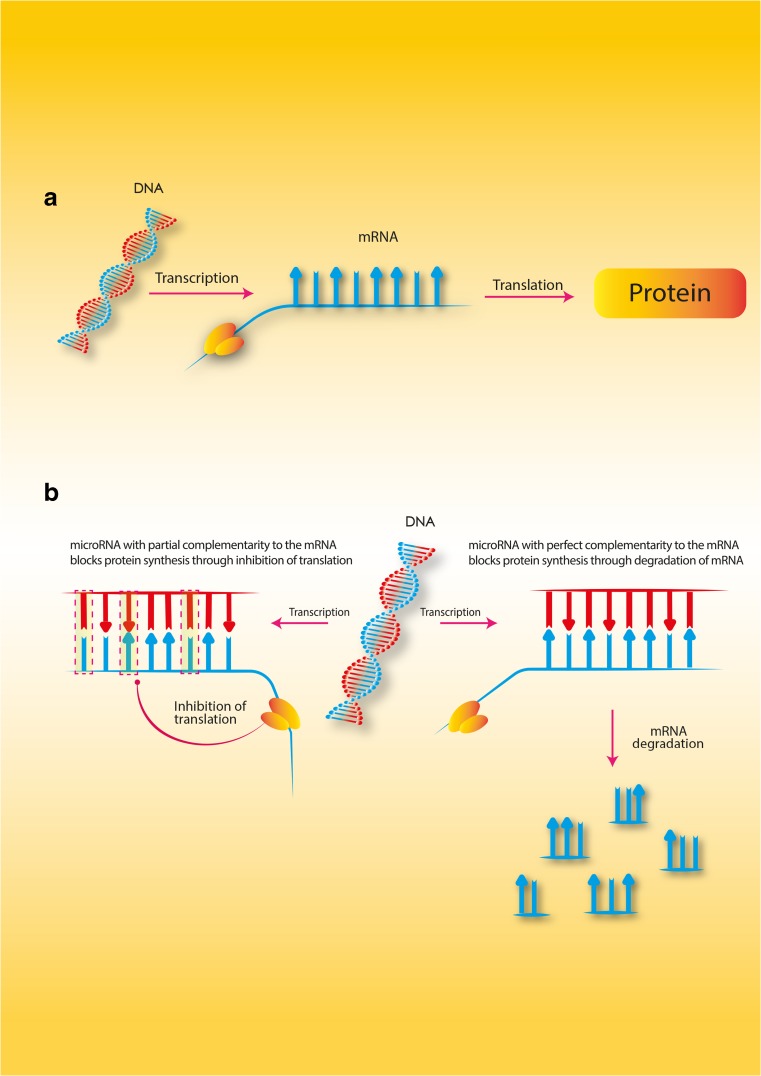
The mechanisms of microRNA action. **a** In normal conditions, without any microRNAs, protein synthesis functions correctly. **b** When microRNAs are present the protein synthesis is blocked by one of two ways. The first process (rarely seen in humans) needs perfect complementarity between the microRNA and the mRNA. When the complex microRNA–mRNA is created, the degradation of mRNA starts, which blocks protein synthesis. The second process (often seen in animal cells) does not need perfect complementarity between the microRNA and the mRNA. In this process, the microRNA binds to mRNA in the 3′UTR and inhibits translation, which blocks protein synthesis

Genes for miRNAs occur in different locations. They can occur in introns and exons of structural genes and in intergenic regions. At the initial formation of an miRNA, the so-called pri-miRNA, is formed with the participation of polymerase II [9, 11, 13]. This pri-miRNA molecule undergoes a process of maturation, in which type III ribonuclease enzymes are involved. These are the Drosha and Dicer enzymes [9, 13–19]. The Drosha enzyme, together with DiGeorge syndrome Critical Region 8 (DGCR8) protein, creates, within the cell nucleus, a nuclear, enzymatic complex called the Microprocessor. As a result of the action of this complex, the RNA molecules are shortened at the 3′ and 5′ ends. Thanks to this, pre-miRNA molecules are formed with a length of around 70 nucleotides. The pre-miRNAs move into the cytoplasm with the help of Exportin-5, where the next stage of their maturation takes place. Within the cytoplasm, maturation takes place principally with the help of the Dicer enzyme and Argonaute proteins (AGO), transactivating response RNA-binding protein (TRBP) and protein activator of PKR (PACT) [9–11, 13, 15, 16, 20–25]. The Dicer enzyme, which recognises a double-stranded pre-miRNA, dissects it around the base of the loop. In this way, double-stranded RNA molecules are formed consisting of around 22 nucleotides, which, with the help of the AGO2 (Argonaute 2 protein), are built into the miRNA-induced silencing complex (miRISC) [9–11, 13–15, 20, 24, 25]. The RISC complex has the ability to recognise the leading RNA duplex strand, which becomes mature miRNA after the degradation of the second strand (Fig. 1). To date, there is only one known example of mature miRNA molecules forming without the Dicer enzyme. This is miR–451, whose precursor pre-miR–451 is an exceptionally short molecule which proceeds through the subsequent stages of maturing to miR–451 without the participation of Dicer [14, 15]. A key element in a mature miRNA molecule is the seven nucleotide fragment at the end of 5′, which ensures binding with the targeted mRNA. Mature miRNAs can have an effect on the level of expression of targeted genes in two ways—through the degradation of mRNA, or by inhibition of translation [11, 13, 24, 26] (Fig. 2). The first process is known as post-transcriptional gene silencing. This requires full complementarity between the miRNA and the targeted mRNA, and very rarely occurs in animal cells. The second process, which typically occurs in animal cells, consists of inhibition of translation and does not depend on full complementarity between the miRNA and the targeted mRNA. In this case, inhibition of gene expression occurs through the attachment of the miRNA to the 3′ untranslated region (3’UTR) of the mRNA, with the participation of a specific protein GW182, which results in inhibition of protein synthesis [13, 24, 26].

The growing interest in miRNAs occurring in humans is due to the role that the molecules play in many important physiological and pathological processes. It has been shown that miRNAs in people take part in the regulation of such processes as the differentiation of haematopoietic stem cells, the differentiation of skeletal muscle cells, neurogenesis, embryogenesis, angiogenesis, insulin secretion, differentiation of mononuclear cells, and the formation and activity of immune system cells [27–30]. Their importance has also been confirmed in conditions such as infections, cancer, metabolic diseases, autoimmune diseases and cardiovascular diseases [13, 31–44].

## MiRNA and left ventricular remodelling

Particularly close attention has been paid recently to some miRNAs due to their participation in the regulation of the function of vascular endothelium and the endothelial cells found in the heart, in addition to their effect on left ventricular remodelling after a myocardial infarction. More and more research is finding proof of the key role miRNAs play in myocardial infarction and in the occurrence of post-infarction left ventricular remodelling. This has been demonstrated by miR–532 among others [45]. Research conducted on cardiac endothelial cells (CECs) in cell culture conditions has shown that CECs without miR–532 demonstrate an increased transformation to the fibroblast phenotype, known as endothelial-to-mesenchymal transition (EndMT). Additionally, CECs with an excessive expression of miR–532 have shown an inhibition of EndMT [45]. It has also been shown that, in mice deprived of the miR–532 gene, changes occur in the structure and function of the heart; the ability of cardiac endothelial cells to proliferate is reduced and the ability to vascularise the myocardium after a myocardial infarction is limited [45]. This is particularly important because insufficient density of capillaries in the myocardium is considered to be the key deciding factor in the development of unfavourable left ventricular remodelling and post-infarction heart failure. Interest in this type of miRNA began with the observation that the protective effect of carvedilol in myocardial infarction patients was related to an increase in the expression of miR–532, and a decrease in the expression of prss23 serine protease. This protein is currently considered as being an important, direct target for the action of miR–532 in the heart [45]. Prss23 is a serine protease which has been shown to be an activator of EndMT. EndMT is a basic process, in which cardiac fibroblasts increase production of extracellular matrix proteins, which intensifies the process of cardiac fibrosis. Inhibition of Prss23 gene expression by miR–532 is therefore crucial for the protective action of this miRNA against unfavourable post-infarction left ventricular remodelling.

The importance of other types of miRNA in myocardial infarction and post-infarction left ventricular remodelling has also been confirmed. One of these is miR–145. It has been shown that the plasma concentrations of miR–145 were significantly lower in patients who had suffered an acute myocardial infarction than in people without coronary disease. Additionally, in a group of myocardial infarction patients, a lower plasma concentration of this particular miRNA was related to significantly higher concentrations of B-type natriuretic peptide and troponin T, and a significantly lower left ventricular ejection fraction [46]. It was also shown that in a group of patients admitted to hospital with an acute myocardial infarction, those patients with a higher number of atherosclerotic coronary arteries had a significantly lower plasma concentration of miR–145 [46].

It was shown that miR–155 is also related to unfavourable post-infarction left ventricular remodelling [47]. It has been discovered that a significantly lower expression of miR–155 during the second post-infarction inflammatory response phase determined the development of unfavourable post-infarction left ventricular remodelling in STEMI patients. This was demonstrated in research in which blood samples for testing miR–155 concentrations were taken in the earlier phase (2nd and 5th day) and in the later phase (6 months) after STEMI [47].

MiR–124 is meanwhile recognised as a cardiomyocyte necrosis marker in acute myocardial infarction. This was confirmed in research performed on patients with acute myocardial infarction and in less than 6 h from the onset of their symptoms [48]. Peripheral blood was taken from these patients for testing at defined points in time, such as: on admission to hospital (time ‘0’), and after 6, 12 and 24 h from the beginning of myocardial infarction symptoms. Apart from miR–124, classic myocardial infarction markers such as troponin I and creatine kinase-brain muscle (CK-MB) were also measured. In this research, a significant increase in miR–124 concentrations in peripheral blood was demonstrated in patients with acute myocardial infarction in relation to healthy people in the control group, with the maximum level of this miRNA appearing in the sixth hour from the beginning of myocardial infarction symptoms. MiR–124 positively correlated with levels of troponin I and CK-MB, reaching its peak level even earlier than troponin I or CK-MB. The high specificity of miR–124 was also confirmed for cardiomyocyte necrosis [48].

In another prospective study on a group of 150 patients with acute myocardial infarction, the prognostic value of group 4 miRNA plasma concentrations was assessed, which were found to influence the expression of genes related to left ventricular remodelling [49]. A panel of miRNAs was taken for evaluation, including miR–16/27a/101/150. The plasma concentrations of miRNAs were measured in the 3–4 days after the myocardial infarction. In addition, the plasma concentration of N-terminal prohormone of brain natriuretic peptide (NT-proBNP) was also noted. The function of the left ventricle was assessed in all patients by echocardiography on the day of release from hospital and again on average 176 days after the myocardial infarction. To assess left ventricular contractility, the wall motion index score (WMIS) was used dividing the left ventricle into 16 segments. The adopted borderline value differentiating between impaired systolic function and preserved left ventricular systolic function was WMIS = 1.2, which corresponds to a left ventricular ejection fraction of 40% [49]. This research showed that measuring a combination of 4 miRNAs at release from hospital allowed for a better prediction of left ventricular post-infarction systolic dysfunction than the clinical prognostic indicators and NT-proBNP alone. The combined measurements of all four miRNAs was best for identifying patients who, under further observation, developed left ventricular systolic dysfunction. Among the four miRNAs measured, the strongest individual relationship to the development of left ventricular systolic dysfunction in the period up to 6 months after the myocardial infarction was shown by miR–27a [49]. What is interesting is, that miR–27a participates in the forming and spreading of many types of cancers, as confirmed in numerous research projects [50–59]. Among the four jointly assessed miRNAs mentioned above, a strong individual relationship to post-infarction left ventricular remodelling was also confirmed for miR–150. This has been shown in research on STEMI patients, in whom there was a confirmed strong negative correlation between the degree of intensity of the left ventricular remodelling and the plasma concentration of miR–150. This research showed that patients who developed post-infarction left ventricular remodelling had a plasma concentration of miR–150 that was two times lower [60]. The mechanisms that underlie this relationship are not fully understood.

Newer research has shown the participation of miR–150 in angiogenesis and ischaemia-induced neovascularisation [31]. These processes are regulated to a large degree by vascular endothelial growth factor (VEGF) and proto-oncogene tyrosine-protein kinase Src (Src), which is necessary for VEGF to function. Src has the ability to activate the phosphoinositide 3-kinase/protein kinase B pathway, which is related to the survival of vascular endothelial cells, their migration and angiogenesis. Src also activates soluble nitric oxide synthase (sNOS), the enzyme engaged directly, among others, in the induction via VEGF of angiogenesis and neovascularisation in conditions of organ ischaemia [31]. MiR–150 inhibits the expression of SRC signalling inhibitor 1 (SRCIN1), which is an inhibitor of Src, the tyrosine kinase necessary for VEGF induced angiogenesis. It has been shown that in conditions of ischaemia, and also in the case of hypercholesterolemia, the activity of SRCIN1 is considerably increased, and hence the activity of Src and endothelial NOS (eNOS) is significantly reduced. In cell culture conditions when vascular endothelium cells were grown and exposed to the action of oxidised LDL cholesterol, it was shown that increase in the expression of miR–150 significantly reduced the expression of SRCIN1, at the same time increasing the activity of Src and eNOS, and intensifying angiogenesis. The proangiogenic action of miR–150 completely ceases after the addition of the specific Src kinase inhibitor [31]. Additionally, ischaemia-induced damage to human cardiomyocytes, evaluated in vitro, turned out to be significantly inhibited by miR–150 [61]. From the point of view of pathogenesis of atherosclerosis as a principal cause of myocardium ischaemia, important results also came out of research which confirmed that miR–150 inhibits the formation of foam cells from macrophages exposed to the action of oxidised LDL–cholesterol molecules [62]. This was confirmed in vitro, demonstrating that miR–150 inhibits the accumulation of molecules of LDL–cholesterol by macrophages and increases their release through these cells by silencing the adiponectin receptor gene (AdipoR2) [62].

Growing interest in miRNAs as biomarkers useful in cardiovascular diseases, is due to their participation in pathophysiological processes related to cardiovascular diseases as well as their stability in blood and in urine [63]. Increasing numbers of research projects aim to isolate the types of miRNA that are most specific for cardiovascular diseases and which are released into the bloodstream during a myocardial infarction. Among the types of miRNA for which such a relationship with cardiovascular diseases has been confirmed, some appear in large amounts both in the myocardium and in the skeletal muscles. Among these are miR–1, miR–133a and miR–499-5p [64, 65]. Other types of miRNA meanwhile only appear in cardiomyocytes. These include miR–208a and miR–208b [12, 65]. The significance of the all these types of miRNA was tested in research, among others, using an animal model of myocardial infarction, in which a myocardial infarction was induced in pigs percutaneously using coronary balloon angioplasty to close the proximal segment of the left anterior descending artery (LAD) [12]. The same research also tested the concentrations of these miRNAs in the blood and urine of patients admitted to hospital with STEMI so that an initial percutaneous coronary intervention (PCI) could be carried out. In the animal model of myocardial infarction, an ischaemia-reperfusion protocol was deliberately used to best mimic the situation that appears in STEMI patients who have undergone PCI. In the animal experiments, a significant increase was found in blood concentrations of miR–1, miR–133a, miR–208b and miR–499-5p resulting from balloon-induced myocardium ischaemia. It must be emphasised, however, that for the whole time the coronary artery was closed by the balloon, no changes were observed in the concentrations of the tested miRNAs. No earlier than 20 min after reperfusion, that is 60 min from the beginning of ischaemia, rapid increases in blood concentrations of miR–1, miR–133a and miR–208b were noted, with peak concentrations in the 120th minute. There was a subsequent sharp drop in such concentrations to 50% of the peak value after a further 30 min. However, a higher concentration of miR–499-5p was sustained for considerably longer, indicating a longer elimination time. No miR–208a was detected [12]. Similar results were obtained in STEMI patients. The blood concentrations of miR–1, miR–133a, miR–208b and miR–499-5p were significantly higher in these patients than in the healthy individuals in the control group, with this rise occurring within 12 h from the beginning of STEMI symptoms [12]. The rise in concentrations of individual miRNAs in comparison to healthy individuals varied, reaching 3000 times more for miR–208b, 300 times more for miR–1, 70 times more for miR–133a and 250 times more for miR–499-5p. High concentrations of miR–208b and miR–499-5p also occurred on the 2nd and 3rd day from the beginning of symptoms. MiR–208a was not detected. This research confirmed the varying dynamics of degradation and excretion of the above-mentioned miRNAs. After 24 h from the beginning of STEMI symptoms, miR–1 and miR–133a were found in patients’ urine, but not miR–208b or miR–499-5p [12]. In terms of the correlation with classic, biochemical and clinical indicators of post-infarction damage to the myocardium, only miR–208b showed a significant correlation with concentrations of troponin T and left ventricular ejection fraction. None of the remaining miRNAs showed such a relationship [12]. Due to the very good correlation between the concentrations of miR–208b and troponin T, which is the gold standard in the diagnosis of cardiomyocyte necrosis, miR–208b is currently considered to be a potential myocardial infarction marker. It exhibits 100% sensitivity and specificity in the diagnosis of myocardial infarction [12, 66, 67]. Due to the strong, negative correlation between the blood concentration of miR–208b during a myocardial infarction and left ventricular ejection fraction, miR–208b is also considered to be a prognostic indicator which permits a prognosis of the degree of post-infarction left ventricular damage and the development of heart failure [12, 67]. This high miR–208b prognostic value, as a parameter allowing for prognosis in myocardial infarction patients of post-infarction left ventricular remodelling and the development of heart failure, has been confirmed in research, in which miR–208b was assessed together with miR–34a [67]. This research confirmed that the combination of these two microRNAs has a greater predictive value in the prognosis of post-infarction left ventricular remodelling and clinically relevant end points 6 months after a myocardial infarction than measuring them each individually [67].

The diagnostic and prognostic significance of miR–1 for post-infarction left ventricular remodelling has also been confirmed in the SITAGRAMI clinical trial patient population [68]. Plasma concentrations of miR–1 were assessed, together with miR–29b, miR–21, and miR–92a, in the fourth and ninth STEMI hour and 6 months after STEMI. Both miR–1, as well as miR–29b and miR–21 showed a similar plasma concentration increase dynamic during a myocardial infarction, with a peak in the 9th hour after a myocardial infarction, as well as concentrations after 6 months equal to those in the fourth hour after the onset of a myocardial infarction. Only miR–92a plasma concentrations did not change significantly during a myocardial infarction. Among the microRNAs tested in this research, only miR–1 and miR–29b had a significant correlation confirmed with unfavourable post-infarction left ventricular remodelling, indicating their usefulness as biomarkers of post-infarction left ventricular remodelling in myocardial infarction patients [68]. As far as the above-mentioned miR–208a is concerned, similar to earlier research, it was not initially detected in the acute phase of a myocardial infarction, both in the animal myocardial infarction model and in patients with STEMI [12]. However, in an experiment using a rat myocardial infarction model, a rise in the miR–208a concentration was confirmed, causing an increase in the expression of myocardial endoglin [69]. Endoglin is a glycoprotein and is a co-receptor for transforming growth factor-beta 1 (TGF–beta 1) and TGF–beta 3 and mediates in the profibrotic effect of angiotension II on cardiac fibroblasts and on the production of the intercellular matrix. This research, using the model of a myocardial infarction in rats, showed that the use of atorvastatin and valsartan after a myocardial infarction decreased myocardial infarction–induced myocardial fibrosis by lowering the expression of miR–208a and myocardial endoglin [69].

The pathophysiological mechanisms relating to the effect of the remaining microRNAs mentioned above are still being researched. One such study confirmed that an increase in the expression of miR–133a in mice with pressure overload of the left ventricle caused by transverse aortic constriction, reduced myocardial fibrosis by inhibiting the expression of connective tissue growth factor (CTGF) [70]. This result is consistent with that obtained in another study using fibroblast cultures, in which it was confirmed that an excessive expression of miR–133 reduced the production of various types of collagen by inhibiting CTGF [71].

It was also shown that there was a significant increase in the expression of miR–184 in the acute phase of a myocardial infarction, with a peak blood concentration reached 24 h after the beginning of myocardial infarction symptoms [72]. This was confirmed in a study in which levels of miR–184 in blood samples taken in successive hours after a myocardial infarction and on the 7th and 14th days after the myocardial infarction, were compared with blood samples taken from patients with stable coronary disease and from healthy volunteers [72]. As early as 6 h from the beginning of symptoms, a significant increase in the level of miR–184 was noted in patients with acute myocardial infarction (AMI) compared to both patients with stable angina and healthy volunteers. After reaching a maximum value within 24 h after the beginning of symptoms, this level started to decrease, returning to normal 7 to 14 days after a myocardial infarction. After this period, the expression of miR–184 in AMI patients was comparable to that found in both control groups. This study also confirmed the existence of a correlation between expression of miR–184 and post-infarction left ventricular remodelling parameters, such as the concentration of NT-proBNP, the left ventricular-end diastolic diameter (LVEDd) and the left ventricular ejection fraction (LVEF), which were assessed before treatment and 14 days after the myocardial infarction. The dynamic of changes of the aforementioned parameters was assessed both before and after treatment using primary PCI. The presence of a significant positive correlation between the level of miR–184 expression and the change in NT-proBNP concentrations before and after treatment, changes in the LVEDd values before and after treatment, and changes in LVEF before and after treatment have been found [72]. Of particular interest in this study is the finding that after 1 year of observation following a myocardial infarction, the level of miR–184 expression was still varied and showed a positive correlation with major adverse cardiac events (MACE). For this reason, we know that a higher level of miR–184 expression exists both in the ischaemia phase and in the reperfusion phase, as well as at the stage of heart repair after a myocardial infarction [72]. Various mechanisms can be considered through which miR–184 can participate in the process of cardiomyocyte apoptosis, left ventricular remodelling and left ventricular muscle fibrosis. One of these is hypoxia-inducible factor-1alpha (HIF-1alpha), which plays an important role in angiogenesis in hypoxic conditions [72].

The crucial importance of miRNAs in the process of cardiomyocyte apoptosis and myocardial fibrosis under hypoxic conditions in myocardial infarction has also been confirmed in research by Yi Sun Song et al. [73]. These researchers looked for miRNAs whose expression levels changed significantly under the influence of transplantation of bone marrow-MSC (BM-MSC) into the area of myocardial infarction. Growing interest in this form of treatment is due to its favourable effects both in animal myocardial infarction models and in humans in terms of a reduction in the area affected by a myocardial infarction, and an improvement in left ventricular function after a myocardial infarction. A reduction in the intensity of apoptosis of cardiomyocytes has been confirmed, as well as a reduction in post-infarction myocardial fibrosis resulting from therapy using various types of MSC. However, the mechanisms of such action are still not fully understood. It has been confirmed that this occurs principally through the paracrine effect of substances released from the MSC and transplanted into the damaged myocardium area, and not through direct regeneration of the myocardium via the MSC. The research demonstrated that the substance released by the MSC is VEGF, which has a strong paracrine effect on inhibiting post-infarction apoptosis of cardiomyocytes and myocardial fibrosis. This study confirmed that VEGF powerfully inhibits the level of expression of miR–23a and miR–92a, which are powerful substances that promote cardiomyocyte apoptosis and myocardial fibrosis. In the rat myocardial infarction model, it was shown that the level of expression of miR–23a and miR–92a significantly rose in the acute phase of a myocardial infarction. Rats which received transplants of BM-MSC in the myocardial infarction area, had a significant rise in the concentration of VEGF, and as a result, a considerable drop in the level of expression of miR–23a and miR–92a when compared to rats with the same myocardial infarction that did not receive the BM-MSC transplant [73]. This animal myocardial infarction model also confirmed substantially less apoptosis and fibrosis in the area of the myocardium in the group of rats which received BM-MSC transplants in the myocardial infarction area, compared to those which were given saline solution. These results were also confirmed in tests in vitro [73]. This research shows that a reduction in the expression of miR–23a and miR–92a due to VEGF plays a crucial role in the favourable, therapeutic effect of BM-MSC transplantation in the rat myocardial infarction model. This forms the basis for researching methods of treating myocardial infarction patients using paracrine substances such as VEGF, which modulate miRNA expression and as a result inhibit apoptosis of cardiomyocytes and myocardial fibrosis.

The effect of miRNAs on the intensification of post-infarction myocardial fibrosis processes was also studied in terms of their effect on expression of TGF–beta1, which is a known mediator of organ fibrosis processes and regulates the function of fibroblasts [74]. It was shown that an increased expression of miR–24 inhibits the expression of TGF–beta1 in cardiac fibroblasts [74]. In a study based on a myocardial infarction model in mice, a substantial decrease was confirmed in miR–24 expression in the acute phase of a myocardial infarction, with the greatest drop 1 week after the myocardial infarction with a return to the starting level 28 days after the myocardial infarction [74]. Decreased miR–24 expression in the acute phase of a myocardial infarction was connected with intensification of heart fibrosis processes mainly in the myocardial infarction region and in the myocardial necrosis border zone. A negative correlation has been shown between miR–24 expression and the amount of type 1 collagen, fibronectin and TGF–beta1 in different areas of the heart in mice with experimentally induced infarctions [74]. This study also confirmed that injecting this area of the myocardium with viral vectors carrying miR–24, limits the area of myocardial infarction and reduces post-infarction scarring. This was confirmed by echocardiographical assessment of left ventricular function carried out both immediately after ligation of LAD and 2 weeks after the procedure. Injection of viral vectors carrying miR–24 into the myocardium caused an improvement in left ventricular fractional shortening (LVFS) and LVEF 2 weeks after the myocardial infarction. This same procedure also resulted in a decrease in the rate of intensity of myocardial fibrosis processes, confirmed by analysis of samples from the myocardial necrosis border zone, as well as after examination of the whole heart removed 2 weeks after the myocardial infarction. In comparison to the control group, a significantly lower expression of type 1 collagen, fibronectin and alpha-smooth muscle cell actin (alpha–SMA) was found in hearts which had undergone miR–24 transfection [74]. This may show that miR–24 inhibits excessive synthesis of the extracellular matrix (ECM), and prevents the transformation of fibroblasts in an infarcted heart 2 weeks after this event.

In cell culture conditions, human cardiac fibroblasts (HCFs) were tested by transfecting them, among others, with miR–125b using plasmid vectors [75]. HCFs serve a very important role both in regular production of extracellular matrix components, as well as in pathological heart fibrosis and post-infarction left ventricular remodelling. It has been confirmed that miR–125b stimulates the proliferation and migration of HCFs, and increases the expression of alpha–SMA and collagen I and II, achieving these effects by decreasing the expression of secreted frizzled-related protein 5 (SFRP5) [75]. Transfecting HCFs with SFRP5 using the same plasmid vectors has the opposite effect to transfecting these cells with miR–125b. As plasma concentrations of miR–125b rise significantly in the acute phase of a myocardial infarction, development of therapies based on effective inhibition of miR–125b expression and/or increasing SFRP5 expression, are potentially an attractive approach to preventing post-infarction fibrosis and left ventricular remodelling [75].

Other research confirmed the influence of miR–17 on increased ECM degradation in the heart during a myocardial infarction. It was shown that the substantial rise in miR–17 expression during a myocardial infarction was connected to significant suppression in the expression of tissue inhibitor of metalloproteinase 1 and 2 (TIMP1/2), which resulted in increased proteolytic matrix metalloproteinase 9 (MMP9) activity and caused an increase in ECM degradation as well as increased post-infarction left ventricular dysfunction [76]. In addition, it was confirmed that antagomir, which blocks the action of miR–17, restores TIMP1/2 activity, thus normalising MMP9 proteolitic activity around the intercellular heart matrix, and improving post-infarction left ventricular function. This study confirmed that, by regulating TIMP1/2 expression, miR–17 plays a key role in maintaining the balance between matalloproteinase activity and their inhibitors within the intercellular matrix in the heart [76]. Disruption of this balance in the course of ischaemia leads to pathological remodelling of the intercellular matrix, which ultimately results in widening of the left ventricle and post-infarction heart failure. This confirmation of the possibility of blocking the action of miR–17 using a specific antagomir and the subsequent improvement in left ventricular function, gives grounds for further research into new therapeutic approaches to protect against post-infarction left ventricular remodelling and heart failure.

## Potential applications and future perspectives

In the authors’ opinion, one early clinical application for the determination of microRNA plasma concentrations may possibly be their use as markers of cardiomyocyte necrosis and as predictors of post-infarction left ventricular remodelling and the development of heart failure. For example, it has been confirmed in clinical trials that miR–24 is a specific marker of cardiomyocyte necrosis, correlating well with classical markers such as troponin I or CK-MB. It was also confirmed that the level of miR–24 myocardial infarction peaks even earlier than that of troponin I [48]. In addition, miR–208b is also considered to be an excellent marker of cardiomyocyte necrosis, with good potential for clinical use in the diagnosis of myocardial infarction. It correlates very well with troponin T. MiR–208b is characterised by 100% sensitivity and specificity in the diagnosis of myocardial infarction [12, 66, 67]. The miR–208b plasma concentration in patients with myocardial infarction may also be a good prognostic indicator of post-infarction left ventricular remodelling and the development of heart failure. Using the combined parameters of miR–208b and miR–34a gives a particularly high predictive value for the prognosis of left ventricular remodelling and the development of heart failure [67]. Under clinical trial conditions, the importance of miR–1 and miR–29b as biomarkers of post-infarction left ventricular remodelling was also confirmed. Determining plasma concentrations of miR–27a and miR–150 may also be clinically useful in patients with myocardial infarction. In patients with STEMI, it was confirmed that measuring these types of microRNA on the day of discharge from the hospital can effectively predict the development of left ventricular systolic dysfunction during long-term observation [49, 60]. Nevertheless, it is important to make clear that larger trials need to confirm the additional benefit of using miRNAs as biomarkers, over those existing biomarkers of the aforementioned cardiovascular pathologies.

However, the most attractive, though more long-term, perspective seems to be in using our knowledge of microRNA in the treatment of patients with cardiovascular diseases. One example is an interesting therapeutic concept based on the experimental data collected so far on the role of miR–125b in stimulating the proliferation and migration of HCF and increasing the expression of alpha–SMA and collagen I and III by reducing the expression of SRP5. The concept is based on the transfection of HCF by SFRPS using plasmid vectors [75]. As the plasma concentrations of miR–125b significantly increase during the acute phase of myocardial infarction, the development of therapies based on the effective inhibition of miR–125b expression and/or increasing expression of SFRP5 in HCF becomes a potentially attractive approach to the prevention of fibrosis and left ventricular remodelling after myocardial infarction.

Another potential, future application of this data about miRNA is to treat patients, in clinical practice, with the use of BM-MSCs. Experiments on myocardial infarction in animals have confirmed a reduction in the intensity of apoptosis of cardiomyocytes and of myocardial fibrosis following myocardial infarction, as a result of therapy using various types of MSCs. It has been confirmed that this is mainly due to the paracrine effect of factors released from MSCs, which have been transplanted in the area of myocardial damage, and not by direct regeneration of the myocardium from BM-MSC. The basic substance of this paracrine activity, released by MSCs, is VEGF, which strongly inhibits the expression of miR–23a and miR–92a, which, in turn, promote apoptosis of cardiomyocytes and myocardial fibrosis. The experimental data, which has been collected, confirms that the reduction in miR–23a and miR–92a expression by VEGF plays a key role in the beneficial therapeutic effect of BM–MSC transplantation in a model of myocardial infarction in animals. This forms the basis for future developments in therapies for patients with myocardial infarction, which could use paracrine factors such as VEGF to suppress the expression of miR–23a and miR–92a, and thus inhibit apoptosis of cardiomyocytes and myocardial fibrosis.

The main problem with the miRNA-based therapies is that a single miRNA controls expression of many genes and changing the expression of a miRNA can cause many different side effects. In terms of solving this problem some new technics have been developed but future studies are still needed. Advanced technologies are also required to effectively deliver specific miRNAs to the designated organs or tissues. So there remains many real challenges to overcome in this field, but physicians should be aware of the potential of introducing miRNA-based therapies into clinical medicine in the future.

## Conclusions

Due to the growing body of data confirming the crucial role of different miRNAs in post-infarction heart remodelling and heart failure, miRNAs can be seen not only as potential diagnostic or prognostic markers, but also as potential therapeutic targets for this group of diseases. The relationships shown so far between different types of miRNA and cardiomyocyte apoptosis processes and myocardium fibrosis open the way for the search for detailed pathogenic mechanisms that are the basis of these relationships. Following on from this, is the search for potential therapies in the field of those cardiovascular diseases, for which there is currently a severe lack of effective treatment.
